# Diverse Microbial Community Profiles of Propionate-Degrading Cultures Derived from Different Sludge Sources of Anaerobic Wastewater Treatment Plants

**DOI:** 10.3390/microorganisms8020277

**Published:** 2020-02-18

**Authors:** Pantakan Puengrang, Benjaphon Suraraksa, Peerada Prommeenate, Nimaradee Boonapatcharoen, Supapon Cheevadhanarak, Morakot Tanticharoen, Kanthida Kusonmano

**Affiliations:** 1 Bioinformatics and Systems Biology Program, School of Bioresources and Technology, and School of Information Technology, King Mongkut’s University of Technology Thonburi, Bangkok 10150, Thailand; 2 Excellent Center of Waste Utilization and Management, Pilot Plant Development and Training Institute, King Mongkut’s University of Technology Thonburi, Bangkok 10150, Thailand; 3 Biochemical Engineering and Systems Biology Research Group, National Center for Genetic Engineering and Biotechnology, National Science and Technology Development Agency at King Mongkut’s University of Technology Thonburi, Bangkok 10150, Thailand; 4 Systems Biology and Bioinformatics Research Group, Pilot Plant Development and Training Institute, King Mongkut’s University of Technology Thonburi, Bangkok 10150, Thailand; 5 School of Bioresources and Technology, King Mongkut’s University of Technology Thonburi, Bangkok 10150, Thailand; 6 National Science and Technology Development Agency, Pathumthani 12120, Thailand; 7 Bioinformatics and Systems Biology Program, School of Bioresources and Technology, King Mongkut’s University of Technology Thonburi, Bangkok 10150, Thailand

**Keywords:** 16S rRNA gene-based sequencing, agro-industrial sludge, anaerobic digestion, microbiome, propionate-degrading cultures

## Abstract

Anaerobic digestion (AD) has been used for wastewater treatment and production of renewable energy or biogas. Propionate accumulation is one of the important problems leading to an unstable system and low methane production. Revealing propionate-degrading microbiome is necessary to gain a better knowledge for alleviation of the problem. Herein, we systematically investigated the propionate-degrading cultures enriched from various anaerobic sludge sources of agro-industrial wastewater treatment plants using 16S rRNA gene sequencing. Different microbial profiles were shown even though the methanogenic activities of all cultures were similar. Interestingly, non-classical propionate-degrading key players *Smithella*, *Syntrophomonas,* and *Methanosaeta* were observed as common prevalent taxa in our enriched cultures. Moreover, different hydrogenotrophic methanogens were found specifically to the different sludge sources. The enriched culture of high salinity sludge showed a distinct microbial profile compared to the others, containing mainly *Thermovirga*, *Anaerolinaceae*, *Methanosaeta*, *Syntrophobactor,* and *Methanospirillum*. Our microbiome analysis revealed different propionate-degrading community profiles via mainly the *Smithella* pathway and offers inside information for microbiome manipulation in AD systems to increase biogas production corresponding to their specific microbial communities.

## 1. Introduction

Biogas is an alternative fuel that can be produced by wastewater treatment under the absence of oxygen, called anaerobic digestion (AD). This process consists of various complex organic degrading sub-processes which are driven by microbial communities [1,2]. Even though the AD system has been considered as a promising solution for wastewater treatment and biogas production, the operational stability in several systems is still poor and yields low biogas production. Various factors have been reported as AD inhibitors causing system instability, such as volatile fatty acids (VFAs), long-chain fatty acids (LCFAs), toxic chemical substances, etc. [3,4]. Many studies have been set up to determine optimal process parameters for gaining high biogas production [5,6,7,8].

The anaerobic digestion process entails four steps: hydrolysis, acidogenesis, acetogenesis, and methanogenesis [9]. During hydrolysis, lipids, proteins, polysaccharides, and soluble organic matter are all degraded, with the final products being further treated through acidogenesis to yield volatile fatty acids (VFAs). The acidogenesis step is followed by acetogenesis, during which the VFAs are digested by acetogenic microorganisms producing a smaller molecule, acetate. The last step is methanogenesis, in which methane is generated. This process involves microorganisms called methanogens, which can be categorized into two groups according to their substrates. Acetoclastic methanogens (AMs) use acetate, while hydrogenotrophic methanogens (HMs) use H_2_/CO_2_ as substrates [10]. Through these AD steps, VFA accumulation often occurs because of the rapid degradation from the acidogenic process and thermodynamically unfavorable degradation [11].

The accumulation of propionic acid, one of the VFAs, has been reported as one of the important reasons for low methane production, as its propagation in the system decreases pH and subsequently inhibits methanogenic activity [6,12]. Enriched cultures of propionic-degrading microorganisms for bioaugmentation have been introduced as a solution to alleviate the acid accumulation, resulting in a more stable system and higher biogas productivity [13,14,15,16]. The technique is the practice of adding a particular microbial culture, which can be grown by using specific substrate as a carbon and energy source, to the unstable AD system for enhancing or boosting process performance. This relies on the fact that the propionate-degrading microbes are a key factor for the improvement of stability and efficiency of anaerobic treatment. Understanding the structure and microbial dynamism of the propionic-degrading communities, including mainly propionate degraders and methanogens, is required to better control and manage the microorganisms for reliability of the treatment systems.

A number of propionate-degrading microbes have been reported, with two main pathways of methylmalonyl Co-A (MMC) and dismutation. The MMC pathway was observed with *Syntrophobacter* sp. and *Pelotomaculum* sp. [17,18], and was mostly reported as a route of classical propionate degradation in AD. The overall reaction is: Propionate^−^ + 3H_2_O → Acetate^-^ + HCO_3_^−^ + H^+^ + 3H_2_; ΔG° = 76.1 kJ/mol [19]. *Methanospirillum* sp. has been found as the main HM, required to maintain H_2_ partial pressure for syntrophic activities with *Syntrophobacter* sp. [20,21,22]. On the other hand, the dismutation pathway was found with *Smithella propionica* which dismutates propionate to acetate and a butyrate through a six-carbon intermediate molecule. The overall equation is: 2Propionate^−^ + 2H_2_O → 3Acetate^-^ + H^+^ + 2H_2_; ΔG° = 48.4 kJ/mol [23,24,25], giving more acetate and less hydrogen per one mole propionate compared to the MMC pathway. The *Smithella* was found as syntrophic-oxidizing bacteria with a number of HMs such as *Methanospirillum* sp. [26] and *Methanoculleus* sp. [27]. However, we believe that all related microbes of the processes have not been completely revealed.

Next-generation sequencing (NGS) technologies have been developed, generating a large amount of genetic sequences allowing culture-independent study of living organisms [28,29,30]. This provides a big advantage to understanding microbial communities as beforehand only a few percent of microorganisms could be studied by cultivation in laboratories. The 16S rRNA gene is a commonly used marker to identify microorganisms from a particular environment using NGS. It has also been applied to explore the AD systems for both lab-scale and full-scale digesters [31,32]. Several microorganisms in the AD process were revealed through NGS-based techniques in different digester conditions [33,34]. To our knowledge, a small number of propionate-degrading community studies have been reported [35,36]. Variation of the communities as a whole system from different wastewater sources have still not been completely revealed. There is a need to extend the investigation of the microorganisms in propionate-degrading microbial communities, providing insight for microbial monitoring and manipulation to control the system stability and prevent failure.

Here, we observed anaerobic propionate-degrading communities via the enriched cultures inoculated from different sources of agro-industrial wastewater treatment plants. The microbiome profiles were investigated using a 16S rRNA-based sequencing approach. Firstly, we investigated the shift of microbiome profiles from inoculum to enrichment stages for revealing propionate-degrading communities. Then, we identified common and unique propionate-degrading microbes among the different sludge sources. We discuss this and conclude with the possible propionate-degrading communities and pathways specific to the original sludge sources.

## 2. Materials and Methods

### 2.1. Microorganisms and Enrichment Process

The propionate-degrading cultures used in this study were enriched from different anaerobic sludge sources. The anaerobic sludge was obtained from six full-scale wastewater treatment plants in Thailand, which treated domestic wastewater (Domestic), fruit juice-processing wastewater (FruitJuice), palm oil mill effluent (PalmOil), starch-processing wastewater (Starch), pig manure waste (PigManure), and seafood-processing wastewater (Seafood). Ten g/L from each sludge was inoculated in a 2-liter reactor-equipped gas counter and mixer at room temperature. To enrich the propionate-degrading cultures, all reactors were fed daily with sodium propionate as the sole carbon source. All reactors were operated for 7 months to increase the organic loading rate (OLR) to 3.0 g chemical oxygen demand (COD)/L/d and the hydraulic retention time (HRT) to 5 days. During the enrichment process, all reactors were evaluated by measuring pH, total volatile acid (TVA), alkalinity, COD reduction, and methane production to control the reactor performance. All enriched cultures were measured for specific methanogenic activity (SMA), using acetic acid as a substrate, with three replications. When operating at propionate loading rate of 3.0 g COD/L/d, the performance of all reactors and the activities of all enriched cultures are shown in Table 1.

### 2.2. Sample Collection and Molecular Analysis

To investigate the microbial communities of the anaerobic sludges obtained from the six full-scale anaerobic digesters (called inoculum) and from the enrichment process (called enriched cultures), DNA from all samples was extracted using DNeasy PowerSoil Kit. The extracted DNA was sequenced with TruSeq PCR-Free library following the manufacturer’s protocol designed for the V3-V4 hypervariable region of the 16S rRNA gene. The universal primers, 319F-CCTAYGGGRBGCASCAG and 806-GGACTACNNGGGTATCTAAT, were utilized. The sequencing was based on the Illumina HiSeq platform generating 250 bases paired-end reads. The obtained 16S rRNA gene-based sequencing data have been deposited at European Nucleotide Archive (ENA) under the accession number ERP113548.

### 2.3. Microbiome Analysis Based on the 16S rRNA Gene Sequences

The microbiome analysis of the enriched propionate-degrading culture was performed using Mothur software (version 1.39.5) [38] including the processes of data preprocessing, operational taxonomic units (OTUs) clustering, taxonomic assignment, and microbial diversity analysis. For the data preprocessing step, sequencing adapter sequences were removed and then paired-end reads were merged into contiguous sequences or contigs. Low-quality sequences which contained ambiguous bases (N), undesired length, off-target amplicon, or ≥ 8-base homopolymer length were discarded. The derived sequences were denoised using a Precluster algorithm to reduce single-base sequencing errors. The UCHIME algorithm [39] was used to remove chimeric sequences. The qualified sequences were then utilized for downstream analyses. The *de novo* OTU clustering was performed using 97% sequence similarity to identify the OTUs. Singletons (OTUs having only one sequence among all samples) were considered as sequencing errors and discarded. SILVA database version 132 [40] was utilized for taxonomic assignment of each OTU. Alpha diversity was measured to estimate sequencing coverage and microbial richness using Good’s coverage and Chao1 indices, respectively. To make comparable microbial profiles, sequence abundances were normalized by a scaling technique based on the number of smallest total sequences among studied samples. OTUs with greater than 1% relative abundance across all samples were displayed in the microbial profiles. For beta-diversity analyses, Bray–Curtis dissimilarities among samples were measured for community comparison and used to visualize the principal coordinate analysis (PCoA) and heatmap. The visualization was performed using R version 3.6.1 (ggplot2 [41] and pheatmap [42] packages). Significant differences of the community profiles were estimated by analysis of similarity (ANOSIM) [43]. Dominant OTUs with greater than 1% relative abundance of each sample were retrieved for the identification of major common and unique organisms in propionate-degrading communities among different sludge sources. OTUs found in at least three out of five samples (excluding the Seafood sample) were reported as common OTUs in propionate-degrading communities.

## 3. Results

### 3.1. A Shift of Microbiome Profiles from Inoculums to Enriched Propionate-Degrading Cultures

**Figure 1:**
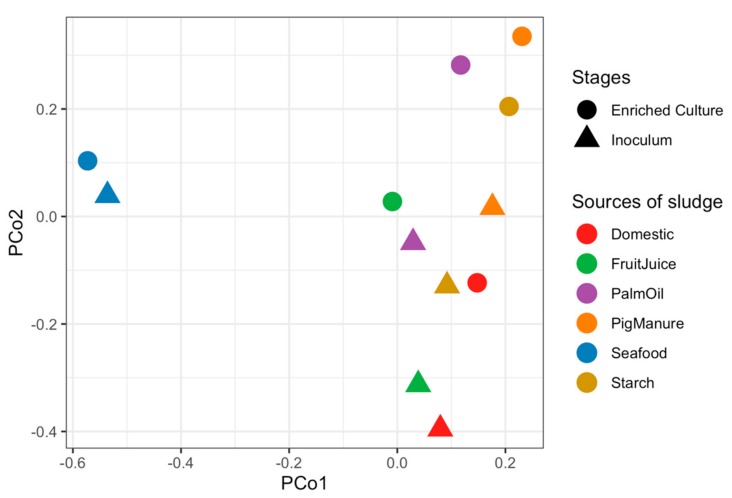
Principal Coordinate Analysis (PCoA) plot showing dissimilar microbial profiles of inoculums and enriched propionate-degrading cultures using the Bray–Curtis measure. Each dot represents an individual anaerobic digestion (AD) sample. Shapes represent stages of the samples: triangles for inoculums and circles for enriched cultures. The colors represent samples from different anaerobic sludge sources.

**Figure 2:**
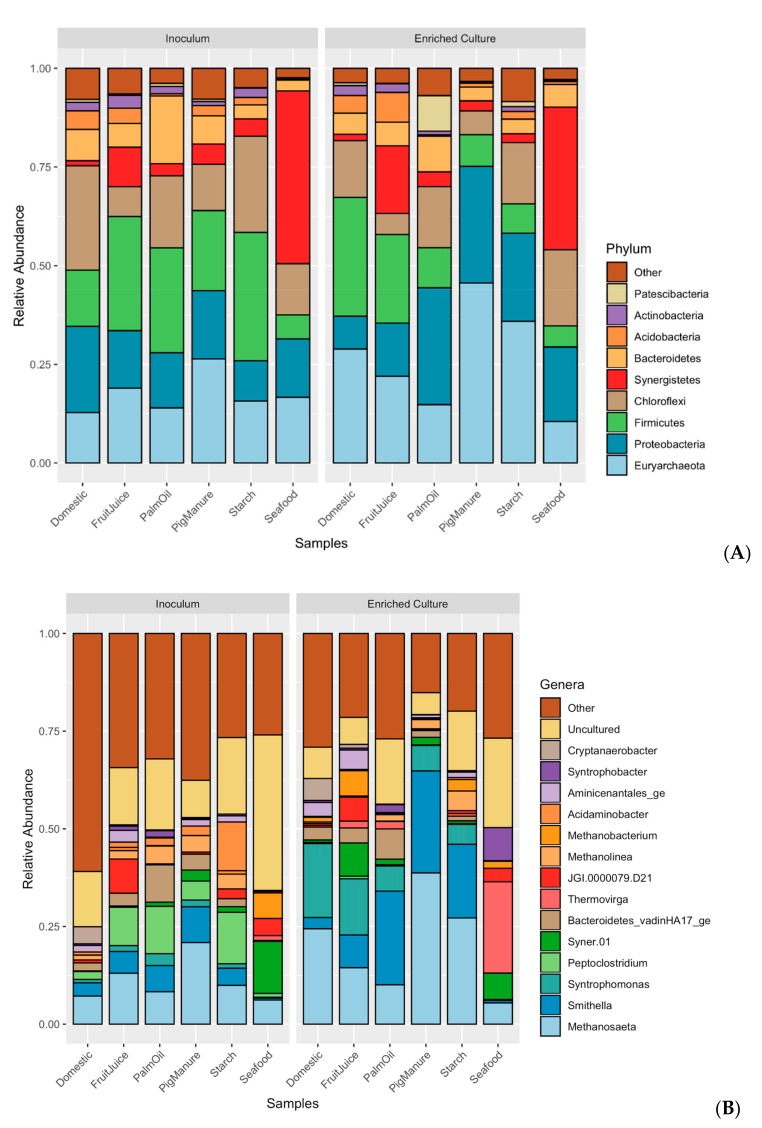
Microbial community profiles showing relative abundance of microbes derived from different anaerobic sludge sources at inoculum and propionate-enriched culture stages, respectively. (**A**) At the phylum level, 19 taxa are dominant (≥1% relative abundance) from the total of 57 assignable phyla. (**B**) At the genus level, 15 dominants of 875 assignable genera are shown.

Microbial communities of sludge inoculums obtained from different full-scale anaerobic wastewater treatment systems and their corresponding propionate-degrading cultures were identified using 16S rRNA gene sequencing. Richness of all samples estimated by Chao1 index vary from 1495.17 to 2811.46 taxa, showing a lower number of enriched cultures than inoculums (Table S1). The similarities of microbial community profiles between the inoculums and enriched propionate-degrading cultures are illustrated via a PCoA plot (Figure 1). Both inoculums and enriched cultures show trends of more similar microbial profiles at the same stages than the same sludge sources, except the Seafood sludge (Tables S2 and S3). The microbial profiles of inoculums are significantly different from the enriched cultures (*p* = 0.04). Figure 2 displays overall taxonomic profiles of all samples with their relative abundance. Nine out of 57 phyla are prevalent, having greater than 1% relative abundance across all samples (Figure 2A). Euryarchaeota, Proteobacteria, Firmicutes, Chloroflexi, and Synergistetes are found as the top five most abundant phyla. These phyla are dominant in both inoculum and enriched cultures, but their proportions are different in each sample. Figure 2B shows the assigned microbial community profiles at the genus level. The overall profiles and dissimilarity measures suggest a shift from inoculum to enriched stages. *Methanosaeta* is a dominant archaeon in all samples of both inoculums and enriched cultures (5.43%–38.72%), but with higher proportion in the enriched cultures. *Smithella*, one of the most abundant bacteria, increased their relative abundance in the enriched samples (0.51%–9.16% in the inoculums and 0.61%–26.09% in the enriched cultures). *Peptoclostridium* show high proportion in the inoculums (0.98%–13.16%) but very less in the enriched cultures (0.06%–0.67%), whereas *Syntrophomonas* have low abundance in the inoculums (0.2%–3.03%) but are mostly prevalent in the enriched cultures (0.22%–18.91%). Particularly, the phylum Synergistetes represents high proportion in the Seafood sludge, distinguishing from other samples in both stages (43.76% and 36.10% at inoculum and enriched culture, respectively). These mainly belong to the genus of *Thermovirga* (1.23% in the inoculum and 23.37% in the enriched culture). In addition, *Syntrophobactor* and *Syner-*01 show high abundance in the enriched sample from the Seafood sludge (8.47% and 6.73%, respectively).

### 3.2. Microbiome Profiles of Propionate-Degrading Cultures Enriched from Different Inoculum Sources

**Figure 3:**
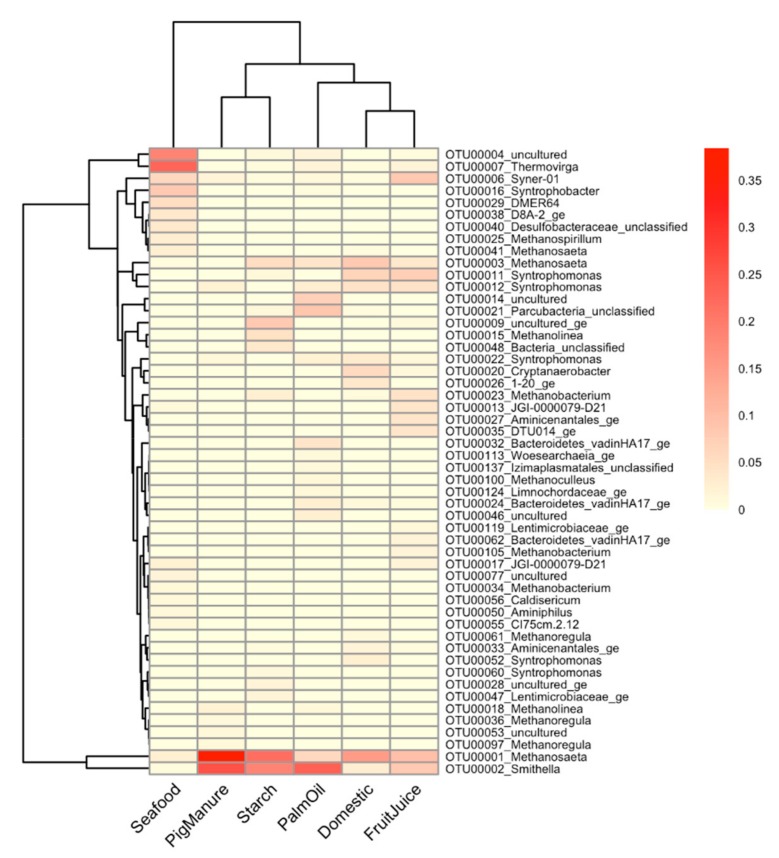
A heatmap shows microbial profiles of the propionate-degrading cultures enriched from different sources. 52 dominant OTUs are presented with their relative abundance. The dendrogram between samples (rows) and OTUs (columns) are drawn based on Bray–Curtis dissimilarity. The OTUs are assigned their taxonomic information at the genus level. The gradient color represents relative abundance of observed OTUs in each sample from low to high as light yellow to red, respectively.

Among the enriched cultures, common and distinct patterns of microbial profiles between different inoculum sources were revealed at the OTU resolution. Figure 3 shows a heatmap of dominant OTUs (greater than 1% relative abundance across all enriched samples) labeled at genus level with their relative abundance. The result reveals 52 dominant OTUs among the propionate-degrading cultures enriched from different inoculum sources from a total of 87 OTUs from both stages (Table S4). The enriched cultures of Domestic and FruitJuice show closet profiles among the six enriched cultures, followed by a pair of PigManure and Starch. *Methanosaeta* (OTU00003) and *Syntrophomonas* (OTU00011 and OTU00012) are commonly dominant in the enriched cultures of Domestic and FruitJuice. The Seafood sample showed the most distinguished profile compared to others. *Methanosaeta* (OTU00001) and *Smithella* (OTU00002) occurred with high abundance in all of the enriched cultures except the Seafood sample (5.86%–38.38% and 2.79%–23.77%, respectively). *Thermovirga* (OTU00007, 22.91%) is remarkable as a unique OTU dominant in the enriched culture from the Seafood sludge. In addition, *Syntrophobacter* (OTU00016, 8.13%), *Desulfobacteraceae* (OTU00040, 3.66%), *Methanospirillum* (OTU00025, 2.71%), and *Methanosaeta* (OTU00041, 2.37%) are also shown with higher abundance in the Seafood sample compared to others. Several unculturable dominant taxa of the class *Anaerolineae* were observed, for example, OTU00004 (18.62%) in the Seafood, OTU00009 (8.18%) and OTU00028 (1.75%) in the Starch, OTU00014 (7.14%) and OTU00046 (2.3%) in the PalmOil, and OTU00053 (1.34%) in the PigManure samples.

### 3.3. Common and Unique Microorganisms in Propionate-Degrading Cultures Enriched from Different Inoculum Sources

We investigated common and unique microorganisms in the propionate-degrading communities among different inoculum sources (Table 2 and Table S5, respectively). Due to the very distinct resulting taxonomic profile of the enriched culture from the Seafood culture compared to other enriched cultures (*p* = 0.018; Table S3), the analysis was performed without the Seafood sample. Table 2 displays common microbes detected among the enriched propionate-degrading cultures (relative abundance greater than 1% in each sample). *Methanosaeta* (OTU00001) and *Smithella* (OTU00002) appeared as common microbes among all enriched cultures. *Syntrophomonas* is also a common genus in all cultures but with different OTUs (OTU00011, OTU00012, and OTU00022). The *Methanosaeta* (OTU00001) was discovered as a main AM. Another *Methanosaeta* (OTU00003) was also found in all enriched cultures except for the Starch sample. Interestingly, different genera of HMs were discovered in each inoculum source (Table 3). For example, *Methanoregula* was found dominantly in the enriched culture of the Domestic (1.20%) and PigManure (2.77%) samples, *Methanobacterium* was found in the FruitJuice sample (6.07%), *Methanolinea* was found in the Starch sample (4.97%), and *Methanoculleus* was found in the PalmOil sample (1.54%). The genus Syner-01 belonging to the family *Synergistaceae* (OTU00006) appears commonly in the enriched cultures of the Domestic, PalmOil and PigManure samples. Furthermore, OTUs of the family *Anaerolineaceae* (OTU00004, OTU00014, and OTU00046) were revealed dominantly in only the enriched culture of the PalmOil sample. Some OTUs were found uniquely in each enriched culture but belong to the same genus of common OTUs such as *Syntrophomonas* in the Domestic sample, etc. (Table S5).

### 3.4. Several Uncultured Microbes Found in the Propionate-Degrading Cultures Using the Culture-Independent Amplicon-Based Sequencing Approach

By performing the 16S rRNA gene sequencing, overall microbial communities of the samples have been revealed without the limitation of cultivation. In our study, the majority of the OTUs could be assigned their taxonomy as well-characterized microbes existing in the public databases (Table S1). However, 8.01% of the identified OTUs were classified as the dominant uncultured microbes at the genus level. These taxa are poorly defined in the available database and annotated as uncultured microbes in different taxonomic levels. Several uncultured microbes were detached in the enriched propionate-degrading communities (Table S6). For example, *Desulfobacteraceae* family was found in the enriched culture of the Seafood sample (1.66%). The class of Anaerolineceae was found with the highest number of OTUs in all the sludge samples, and dominant in several sludge sources such as Seafood, Starch, PalmOil, and PigManure (18.62%, 9.94%, 9.54%, and 1.34%, respectively). In addition, the Seafood samples contain high percent abundance of uncultured microbes in both the inoculums (39.29%) and the enriched cultures (22.89%) (Table S7).

## 4. Discussion

### 4.1. The Schematic Propionate-Degrading Pathway in the Enriched Cultures for Methane Production

**Figure 4:**
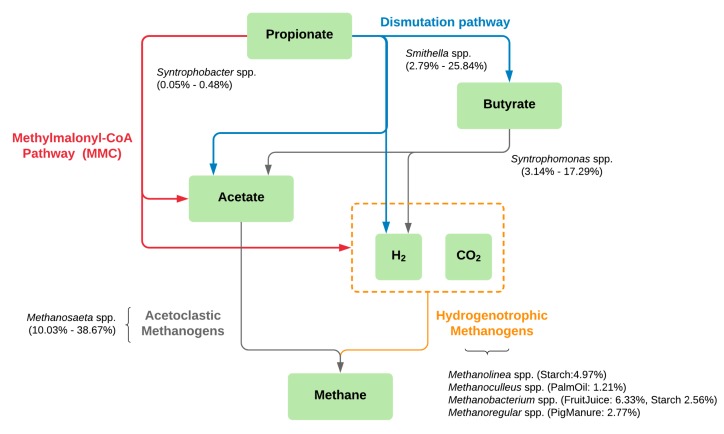
Schematic pathway of methane production based on propionate-degrading cultures enriched from different anaerobic sludge sources excluding the Seafood sample. Colors represent pathways of propionate degradation to methane production; red: methylmalonyl-CoA (MMC) pathway, blue: dismutation pathway, gray: acetoclastic pathway, and yellow: hydrogenotrophic pathway. Microbial taxa found in our study were drawn along the pathways with their percent relative abundance.

With the limited carbon source of only propionate in the enriched cultures, microbial diversities in the samples were lower than in the inoculum sludges (Table S1). The discovered microbial community profiles and their degradation processes could be affected by the single carbon source feeding. Excluding the Seafood sample, our experiment revealed very small percentages of *Syntrophobacter* (<0.5%), which was previously proven as a propionate-degrading bacterium and found in most of the propionate-degrading communities along with HMs [22,35,44,45]. Interestingly, *Smithella* was found to be the dominant propionate-degrading bacterium [26] in our experiment, instead of the regular *Syntrophobacter*. There might be two main reasons for the presence of *Smithella* in the enriched cultures: (1) the nature of the original sludge containing a higher number of *Smithella* than *Syntrophobacter* (Figure 2; Table S4) and (2) *Syntrophobacter* prefers to grow with propionate and sulfate in the medium [23], which corresponds to our experiment that fed the medium without adding sulfate. The results suggest that the main reaction of the propionate degradation (Figure 4 and Table S8) is through *Smithella,* which can produce acetate and butyrate via a six-carbon intermediate, called the dismutation pathway [23,24,25]. The total reactions produced more acetate molecules compared to the classical pathway which belongs to *Syntrophobacter* and *Pelotomaculum* [23]. Following this theoretical perspective, we observed a higher abundance of *Methanosaeta*, which produces methane by acetate degradation, in the enriched samples [46]. Furthermore, *Syntrophomonas* was observed in several enriched samples. It was reported as a butyrate utilizer to produce acetate for AMs in the AD system [47]. Therefore, our studies suggest multi-trophic interaction of *Smithella* that can degrade propionate directly to acetate and convert propionate to butyrate, which is a substrate for *Syntrophomonas* (Figure 4). Consequently, *Methanosaeta* utilizes the resulting acetate from both organisms to produce methane and functions as a key AM in the enriched cultures.

### 4.2. Different Taxa of Hydrogenotrophic Methanogens Found Specifically to Different Sludge Sources

While a single genus of AM was found as dominant taxa in all enriched samples, various genera of HMs were found particular to different sludge sources (Table 3 and Figure 4). In this study, *Methanobacterium*, *Methanoculleus*, and *Methanolinea,* were found in the FruitJuice, PalmOil, and Starch samples, respectively. Different OTUs of *Methanoregula* were found in the Domestic and PigManure samples. All of these HMs were reported in various mesophilic environments [48,49], and some of them, e.g., *Methanolinea* and *Methanoculleus,* were isolated from propionate-enrichment cultures as prevalent methanogen [50,51]. Although relatively smaller amounts of these HMs compared to AMs have been observed, they could also play a role in our systems for methane production by conversion of CO_2_/H_2_. These small amounts could also result from less H_2_ produced from the dismutation pathway compared to the MMC pathway (Table S8). The observed HMs could refer to the syntrophic contribution of propionate degradation with *Smithella* [23]. Several types of HMs resulting from different wastewater treatment sludges suggest possible various pairs of syntrophic propionate oxidation and methane production between *Smithella* and HMs. The information of specific microbial taxa or communities of propionate degradation could be used as a guideline for microbial management, leading to efficient biogas production.

### 4.3. Unique Microbial Community in the Propionate-Degrading Culture Enriched from Seafood Sludge

The Seafood sludge revealed statistically distinct microbial profiles compared to the other sludges from different wastewater sources (Figure 2 and Table S3). *Thermovirga* and *Anaerolineaceae* uncultured groups affiliating to phylum Synergistetes and Chloroflexi, respectively, were found as prevalent organisms in the enriched propionate-degrading culture. *Thermovirga* were reported as amino acid degrading bacteria and were found dominantly in high salinity environments [52]. This is consistent with the condition of the Seafood sample, that originally contained high salinity. *Anaerolineaceae* were found in the AD system relating to granular formation and maintenance [53]. Both *Thermovirga* and *Anaerolineaceae* have been revealed dominantly with *Methanosaeta* in several AD experiments [54,55,56], suggesting that these microbes would play an important role in propionate degradation and biogas production pathways. *Syntrophobacter* and *Methanospirillum* were found as syntrophic propionate-oxidizing bacteria and H_2_-utilizing methanogen, respectively [22,35]. These microbes have relatively higher abundance in the Seafood sample compared to the other five samples, suggesting an observation of the classical MMC pathway instead of our main discovered *Smithella* pathway (Figure 4). Furthermore, the Seafood sample showed the highest HM:AM ratio compared to other samples (Table S9). This corresponds to the result of a higher percent methane production but less SMA, indicating AM activities of utilizing acetates as substrates, compared to other samples (Table 1). The result suggested that the HMs would play more of a role in this sample as the MMC pathway provides more H_2_ than the dismutation pathway (Table S8). The result showed that the Seafood sample has a unique profile and could be further investigated for the enrichment of methanogenic propionate degradation in a saline environment.

### 4.4. Overall Microbial Profiles of Propionate-Degrading Cultures and Unculturable Microbes Revealed Through Amplicon-Based Sequencing

The utilization of NGS allows the study of microbes taken directly from the samples without cultivation, showing all existing microbes with their abundance in the studied sample. Beforehand, a small number of known microbes has been studied, limited by cultivation [22,26,57]. Since microbes live as a community, this high-resolution technique provides a great opportunity to derive an overall picture of a microbial community and provides more insights to understand the dynamism of the studied consortium. In this study, a set of propionate-degrading communities was revealed according to their original sludge sources. Many OTUs of the class Anaerolineae were empirically revealed as predominant uncultured microbes in the enriched propionate-degrading cultures (Table S6). This microbe has been discovered dominantly in several AD systems [56]. In addition, Mcllroy S.J. et al. [56] reported a member of Anaerolineae co-located with *Methanosaeta* spp., which was discovered in our study as major archaea. The function of the Anaerolineae and its synergistic relationship to *Methanosaeta* could be worth further investigation. The information from high-throughput sequencing provided a whole microbial community leading to better understanding of the control and management of the AD systems, as the microorganisms work together in the process.

## 5. Conclusions

The microbiome of the propionate-degrading communities enriched from different inoculum sources was investigated using 16S rRNA gene sequencing analysis. Interestingly, we found *Smithella* as the dominant propionate-degrading bacteria in most of the studied samples, suggesting the dismutation pathway of propionate degradation instead of the classical MMC pathway. The experiment supported a key role of *Smithella* and *Syntrophomonas* that implied a multi-trophic interaction of these two microorganisms to convert propionate to acetate and butyrate, and butyrate to acetate, respectively. A major abundance of *Methanosaeta* was observed as a main methanogen using acetate, while dominant HMs were found specific to different inoculum sources. The Seafood sludge sample shows a distinctive microbial profile containing *Thermovirga*, *Anaerolinaceae,* and *Methanosaeta* as dominant taxa, as well as *Syntrophobacter* and *Methanospirillum* which are mostly reported as regular syntrophic propionate-degrading culture through the MMC pathway. The highest HM:AM ratio was found in the Seafood sample, which corresponds to the MMC pathway producing more hydrogen that is utilized by HMs than the *Smithella* pathway. On the other hand, the relative abundances of AMs in the samples with the dismutation pathway were higher than in the Seafood sample, as more acetates are produced from that pathway. Furthermore, several uncultured bacteria of the class Anaerolinea were revealed in the enriched cultures. Our study shows that digesters with comparable performance and methane production could contain different communities of propionate-degrading microbes corresponding to their original sludge sources. The result suggests that inside information of specific propionate-degrading communities could be further applied to microbial monitoring and manipulation of wastewater treatment systems to increase biogas production.

## Figures and Tables

**Table 1:** Performance of six reactors operating at propionate loading rate of 3.0 g COD/L/d and microbial activities of enriched propionate-degrading cultures.

Anaerobic Sludge from Various Anaerobic Wastewater Treatment Plants	Reactor Performance					Specific Methanogenic Activity (SMA) (g COD/g VSS/d)
	pH	TVA/Alkalinity	COD Reduction (%)	Biogas Composition (%)		
				%CH_4_	%CO_2_	
**Domestic**	7.50	0.30	86.5	60.0	35.5	0.22 ± 0.016
**FruitJuice**	7.50	0.30	85.0	62.5	34.0	0.17 ± 0.011
**PalmOil**	7.49	0.30	86.5	75.0	22.0	0.20 ± 0.009
**Starch**	7.56	0.25	90.0	73.5	23.5	0.22 ± 0.007
**PigManure**	7.57	0.27	89.0	75.5	21.0	0.28 ± 0.003
**Seafood**	7.52	0.35	80.0	80.0	17.5	0.14 ± 0.015

Remark: 1 mole propionate gives 1.75 mole methane and 1.25 mole carbon dioxide [37]. TVA: total volatile acid, COD: chemical oxygen demand.

**Table 2:** A list of common propionate-degrading taxa enriched from different anaerobic agro-industrial sludge sources.

OTUs	Taxonomic Lineage	Propionate-Enriched Culture				
		Domestic	FruitJuice	PalmOil	PigManure	Starch
OTU00001	Archaea; Euryarchaeota; Methanomicrobia; Methanosarcinales; Methanosaetaceae; Methanosaeta	✓	✓	✓	✓	✓
OTU00002	Bacteria; Proteobacteria; Deltaproteobacteria; Syntrophobacterales; Syntrophaceae; Smithella	✓	✓	✓	✓	✓
OTU00003	Archaea; Euryarchaeota; Methanomicrobia; Methanosarcinales; Methanosaeta; Methanosaeta	✓	✓	✓	✓	
OTU00006	Bacteria; Synergistetes; Synergistia; Synergistales; Synergistaceae; Syner-01	✓		✓	✓	
OTU00011	Bacteria; Firmicutes; Clostridia; Clostridiales; Syntrophomonadaceae; Syntrophomonas	✓	✓			✓
OTU00012	Bacteria; Firmicutes; Cloastridia; Clostridiales; Syntrophomonadaceae; Syntrophomonas	✓	✓	✓	✓	
OTU00022	Bacteria; Firmicutes; Clostridia; Clostridiales; Syntrophomonadaceae; Syntrophomonas	✓	✓	✓	✓	

**Table 3:** A list of unique hydrogenotrophic methanogens in propionate-degrading cultures enriched from anaerobic agro-industrial sludge sources.

OTU	Hydrogenotrophic Methanogen		Observed Sample
	Family	Genus	
OTU00061	*Methanoregulaceae*	*Methanoregula*	Domestic
OTU00023	*Methanobacteriaceae*	*Methanobacterium*	FruitJuice
OTU00105	*Methanobacteriaceae*	*Methanobacterium*	FruitJuice
OTU00100	*Methanomicrobiaceae*	*Methanoculleus*	PalmOil
OTU00036	*Methanoregulaceae*	*Metanoregula*	PigManure
OTU00097	*Methanoregulaceae*	*Metanoregula*	PigManure
OTU00015	*Methanoregulaceae*	*Methanolinea*	Starch

## Supplementary Materials

The following are available online at https://www.mdpi.com/2076-2607/8/2/277/s1, Table S1: Alpha-diversity estimation of microbiome in inoculums and their corresponding propionate-degrading cultures, Table S2: Statistical comparison of microbial profile between stages of inoculum and enriched culture using analysis of similarity (ANOSIM), Table S3: Statistical comparison of microbial profiles among different sources of anaerobic sludge using analysis of similarity (ANOSIM), Table S4: Relative abundance of 87 dominant OTUs (relative abundance greater than 1% in at least one sample) in our studied samples, Table S5: A list of unique propionate-degrading microbes enriched from different anaerobic sludge sources, Table S6: A list of SILVA-annotated uncultured microbes found dominantly (greater than 1% relative abundance) in the propionate-degrading cultures, Table S7: Percent relative abundance of uncultured microbes found in the inoculums and propionate-degrading cultures using the culture-independent amplicon-based sequencing approach, Table S8: Reactions of syntrophic metabolism of obligate proton-reducing acetogens and methanogens [19,25]. Table S9: Relative abundance of dominant methanogens found in the inoculum and propionate-degrading cultures and percentage HM:AM ratio.
